# A new species of *Orobdella* (Hirudinida, Arhynchobdellida, Orobdellidae) from Taipei, Taiwan

**DOI:** 10.3897/zookeys.207.3334

**Published:** 2012-06-11

**Authors:** Takafumi Nakano, Yi-Te Lai

**Affiliations:** 1 Department of Zoology, Graduate School of Science, Kyoto University, Kyoto 606-8502, Japan; 2 Institute of Zoology, National Taiwan University, No. 1, Roosevelt Road, Section 4, Taipei 106, Taiwan; 3 Department of Biology, University of Eastern Finland, P. O. Box 111, FI 80101 Joensuu, Finland

**Keywords:** Hirudinida, Orobdellidae, *Orobdella*, new species, first record, gastroporous, Taiwan

## Abstract

A new quadrannulate species of *Orobdella*, *Orobdella ketagalan* **sp. n.**, from Taipei, Taiwan, is described. This is the first record of *Orobdella* and the family Orobdellidae from Taiwan. This new species possesses small, paired sperm duct bulbs in the male reproductive system. In addition to these bulbs, the following combination of characters distinguishes this new species from other quadrannulate species: somite IV uniannulate, male gonopore at XI b6, female gonopore at XIII a1, 1/2 + 4 + 1/2 between gonopores, simple tubular gastroporal duct, lacking epididymides, and undeveloped atrial cornua. Phylogenetic analyses using nuclear 18S rDNA and histone H3 as well as mitochondrial COI, 12S rDNA, tRNA^Val^, and 16S rDNA markers showed that *Orobdella ketagalan* is related to the two Ryukyu Archipelago species *Orobdella dolichopharynx* Nakano, 2011 and *Orobdella shimadae* Nakano, 2011.

## Introduction

Species of the genus *Orobdella* Oka, 1895 are large annelids that feed on earthworms. They are usually 10–20 cm in length (except for *Orobdella koikei* Nakano, 2012, approx. 5 cm) and they inhabit the banks of mountain streams in East Asia (Nakano 2012a, Oka 1895). The systematic position of the genus *Orobdella* has been contentious. *Orobdella* was initially included in the family Gastrostomobdellidae along with the Southeast Asian terrestrial macrophagous leech genus *Gastrostomobdella* Moore, 1929 (Richardson 1971, Sawyer 1986). Although Sawyer (1986) placed Gastrostomobdellidae under Hirudiniformes, recent molecular phylogenetic studies reclassified the family under Erpobdelliformes (Nakano et al. 2012, Oceguera-Figueroa et al. 2011). Nakano et al. (2012) split Gastrostomobdellidae into two families based on phylogenetic analyses as well as morphological discontinuity, and the monotypic family Orobdellidae was erected for *Orobdella*.

Taxonomic and inventory studies on *Orobdella* have progressed recently, and this genus now includes ten species (Nakano 2010, 2011a, b, 2012a, b, in press, Oka 1895, Richardson 1975). *Orobdella* leeches exhibit various types of mid-body somite annulation; three types have been documented thus far: quadrannulate, sexannulate, and octannulate (Sawyer 1986). The quadrannulate mid-body somite is a plesiomorphy of this genus, and the sexannulate form is considered to have evolved in parallel (Nakano 2012a, b, Nakano et al. 2012).

All of the known *Orobdella* species have been described based on specimens collected from Japan, and eight of the ten species have been reported only from Japanese islands (Sawyer 1986). Outside Japan, *Orobdella whitmani* Oka, 1895, which is the type species of the genus, has been recorded from Primorsky Krai, Russia (Gilyarov et al. 1969). However, Nakano (2012a) noted that this specimen from Russia was misidentified as *Orobdella whitmani*, and that it should be considered a new, undescribed species. In addition, *Orobdella tsushimensis* Nakano, 2011 was recently collected from Gageodo Island, Korea (Nakano and Seo in press). In Taiwan, no studies have investigated the species diversity of terrestrial macrophagous leeches. Taiwanese leech species were catalogued by Lai and Chen (2010), but *Orobdella* leeches were not included. Recently, quadrannulate *Orobdella* specimens were collected from Taipei, Taiwan. These materials clearly differ from the other known quadrannulate *Orobdella* species. Therefore, in the present study, *Orobdella* leeches from Taipei are described as a new species. This is the first record of orobdellid leeches from Taiwan. In addition, their phylogenetic position is estimated using nuclear 18S and histone H3 (H3) and mitochondrial COI, 12S, tRNA^Val^, and 16S rDNA (12S–16S) sequence data.

## Materials and methods

**Figure 1.**
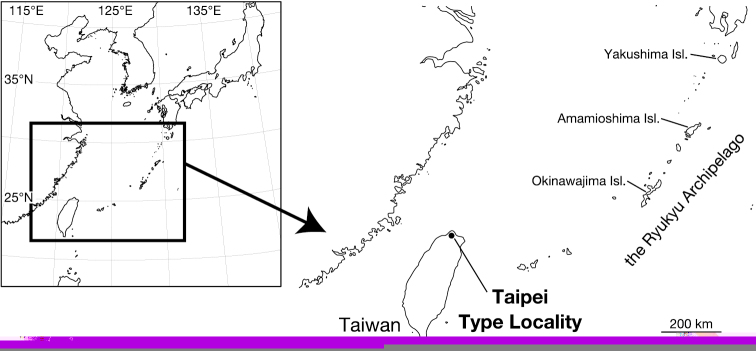
Map showing the collection localities in this study.

Leeches were collected from Taipei, Taiwan (Fig. 1). Botryoidal tissue was taken from specimens, which were fixed in ethanol, for DNA extraction. All of the specimens were preserved in 70% ethanol. Two measurements were taken: body length (BL) from the anterior margin of the oral sucker to the posterior margin of the caudal sucker, and maximum body width (BW). Examination, dissection, and drawings of the specimens were accomplished under a stereoscopic microscope with a drawing tube (Leica M125). The specimens have been deposited in the Zoological Collection of Kyoto University (KUZ).

We used the numbering convention of Moore (1927): body somites are denoted by Roman numerals, and annuli in each somite are given alphanumeric designations.

The extraction of genomic DNA followed (Nakano 2012a). The primer sets used in this study are listed in Table 1: for 18S, A and L, C and Y, and O and B (Apakupakul et al. 1999) were used; for H3, H3aF and H3bR (Colgan et al. 1998); for COI, LCO 1490 and HCO 2198 (Folmer et al. 1994), and LCO-in and HCO-out (Nakano 2012a); for 12S–16S, 12SA-in and 12SB-out (Nakano 2012a). The DNA sequencing methods for the above four markers followed Nakano (2012a). The following DNA sequences were newly obtained and deposited in GenBank (Table 2): 18S and H3 sequences from the holotype (KUZ Z208) of the new species, and COI and 12S–16S sequences from the holotype (KUZ Z208) and three paratypes (KUZ Z209–Z211) of the new species. The DNA sequences of the holotype (KUZ Z208) were analyzed in the following phylogenetic analyses. The other sequences were taken from GenBank (Table 2). For the outgroup, three Erpobdelliformes leeches were included in the phylogenetic analyses: *Erpobdella japonica* Pawłowski, 1962 (Erpobdellidae), *Gastrostomobdella monticola* Moore, 1929 (Gastrostomobdellidae), and *Mimobdella japonica* Blanchard, 1897 (Salifidae).

**Table 1.** PCR and cycle sequencing (CS) primers used in this study. Sources: ^a^Apakupakul et al. (1999), ^b^Colgan et al. (1998), ^c^Folmer et al. (1994), ^d^Nakano (2012a).

**Gene**	**Primer name**	**Reaction**	**Primer sequence (5’ → 3’)**
18S			
1	A^a^	PCR & CS	AACCTGGTTGATCCTGCCAGT
	L^a^	PCR & CS	CCAACTACGAGCTTTTTAACTG
2	C^a^	PCR & CS	CGGTAATTCCAGCTCCAATAG
	Y^a^	PCR & CS	CAGACAAATCGCTCCACCAAC
3	O^a^	PCR & CS	AAGGGCACCACCAGGAGTGGAG
	B^a^	PCR & CS	TGATCCTTCCGCAGGTTCACCT
Histone H3			
	H3aF^b^	PCR & CS	ATGGCTCGTACCAAGCAGACVGC
	H3bR^b^	PCR & CS	ATATCCTTRGGCATRATRGTGAC
COI			
1	LCO1490^c^	PCR & CS	GGTCAACAAATCATAAAGATATTGG
	HCO2198^c^	CS	TAAACTTCAGGGTGACCAAAAAATCA
2	LCO-in^d^	CS	TCCAGAACGTATTCCATTATTTG
	HCO-out^d^	PCR & CS	TCTGGGTAGTCAGAATATCG
12S–16S			
	12SA-in^d^	PCR & CS	AATTAAAACAAGGATTAGATACCC
	12SB-out^d^	PCR & CS	AACCCATAATGCAAAAGGTAC

**Table 2.** Samples used for the phylogenetic analyses. Information on vouchers, collection localities, and GenBank accession numbers is provided.UNIMAS, the Universiti Malaysia Sarawak. Sources: ^a^Nakano (2012a), ^b^Nakano (2012b), ^c^Nakano et al. (2012).

**Species**	**Voucher**	**18S**	**Histone H3**	**COI**	**12S–16S**
*Orobdella ketagalan* sp. n.	KUZ Z208 Holotype	AB704785	AB704786	AB704787	AB704788
*Orobdella ketagalan* sp. n.	KUZ Z209 Paratype			AB704789	AB704790
*Orobdella ketagalan* sp. n.	KUZ Z210 Paratype			AB704791	AB704792
*Orobdella ketagalan* sp. n.	KUZ Z211 Paratype			AB704793	AB704794
*Orobdella esulcata*	KUZ Z29 Holotype	AB663655^c^	AB698873^b^	AB679664^a^	AB679665^a^
*Orobdella dolichopharynx*	KUZ Z120 Holotype	AB663665^c^	AB698876^b^	AB679680^a^	AB679681^a^
*Orobdella ijimai*	KUZ Z110 Topotype	AB663659^c^	AB698877^b^	AB679672^a^	AB679673^a^
*Orobdella kawakatsuorum*	KUZ Z167 Topotype	AB663661^c^	AB698878^b^	AB679704^a^	AB679705^a^
*Orobdella koikei*	KUZ Z156 Holotype	AB698883^b^	AB698882^b^	AB679688^a^	AB679689^a^
*Orobdella mononoke*	KUZ Z224 Holotype	AB698868^b^	AB698869^b^	AB698866^b^	AB698867^b^
*Orobdella octonaria*	KUZ Z181 Topotype	AB698870^b^	AB698871^b^	AB679708^a^	AB679709^a^
*Orobdella shimadae*	KUZ Z128 Holotype	AB663663^c^	AB698875^b^	AB679676^a^	AB679677^a^
*Orobdella tsushimensis*	KUZ Z134 Holotype	AB663653^c^	AB698872^b^	AB679662^a^	AB679663^a^
*Orobdella whitmani*	KUZ Z45 Topotype	AB663657^c^	AB698874^b^	AB679668^a^	AB679669^a^
*Erpobdella japonica*	KUZ Z178	AB663648^c^	AB698879^b^	AB679654^a^	AB679655^a^
*Gastrostomobdella monticola*	UNIMAS/A3/BH01/10	AB663649^c^	AB698880^b^	AB679656^a^	AB679657^a^
*Mimobdella japonica*	KUZ Z179	AB663650^c^	AB698881^b^	AB679658^a^	AB679659^a^

H3 and COI sequences were aligned by eye because there were no indels. Nuclear 18S and mitochondrial 12S–16S sequences were aligned using MAFFT X-INS-I (Hofacker et al. 2002, Katoh and Toh 2008, McCaskill 1990, Tabei et al. 2008) taking into account RNA secondary structure information, and then refined with GBLOCKS (Castresana 2000). Aligned sequences of 18S was 1787 bp in length; those of H3, COI, and 12S–16S were 327, 1266, and 410 bp, respectively. The concatenated sequences thus yielded a total of 3790 bp positions.

Phylogenetic trees were constructed using maximum likelihood (ML) and Bayesian inference (BI). ML phylogenies were calculated using TREEFINDER v October 2008 (Jobb et al. 2004) with the tool package Phylogears v 2.0 (Tanabe 2008), and then non-parametric bootstrapping (Felsenstein 1985) was conducted with 500 replicates. The best-fit models for each partition were selected using the Akaike information criterion (Akaike 1974) by using Kakusan 4 (Tanabe 2011): for 18S, the Jobb 2008 model (J2) with gamma distribution (+G) and proportion of invariant sites (+I) was selected; for H3 1st position, the Tamura-Nei model (TN93); for H3 2nd position, the Jukes-Cantor model (JC69); for H3 3rd position, J2+G; for COI 1st position, TN93+G+I; for COI 2nd position, the transversion model (TVM)+I; for COI 3rd position, TN93+G; for 12S, the general time reversal model (GTR)+G; for tRNA^Val^, the Hasegawa-Kishino-Yano model (HKY85)+G; and for 16S, the transition model (TIM)+G. BI and Bayesian posterior probabilities (BPPs) were estimated using the MPI version of MrBayes v 3.1.2 (Altekar et al. 2004, Huelsenbeck et al. 2001, Ronquist and Huelsenbeck 2003). The best-fit models for each partition were identified using the Bayesian information criterion (Schwarz 1978) also by using Kakusan 4: for 18S, the Kimura 1980 model (K80)+I; for H3 1st and 2nd positions, JC69; for H3 3rd position, HKY85+G; for COI 1st position, GTR+G+I; for COI 2nd position, the Felsenstein 1981 (F81) model+I; for COI 3rd position, HKY85+G; for 12S, GTR+G; and for tRNA^Val^ and 16S, HKY85+G. Two independent runs of four Markov chains were conducted for 20 million generations and the tree was sampled every 100 generations. The parameter estimates and convergence were checked using Tracer v 1.5 (Rambaut and Drummond 2009), and based on the results the first 50,001 trees were discarded.

Nodes with bootstrap (BS) values higher than 70% were considered sufficiently resolved (Hillis and Bull 1993). Nodes with BPPs higher than 95% were considered statistically significant (Leaché and Reeder 2002).

## Taxonomy

### Family Orobdellidae Nakano, Ramlah & Hikida, 2012

urn:lsid:zoobank.org:act:5F5BABE8-BD26-4FC7-9593-F73E62E26122

Genus *Orobdella* Oka, 1895

urn:lsid:zoobank.org:act:FA8333ED-8C17-41FD-AFC1-62A4F98D4AC1

#### Orobdella ketagalan sp. n.

urn:lsid:zoobank.org:act:AFF291DF-E13F-46A3-A965-14B92E23F520

http://species-id.net/wiki/Orobdella_ketagalan

Figs 2 [Fig F3] –4

##### Diagnosis.

Somite IV uniannulate, somites VIII–XXV quadrannulate. Pharynx reaching to posterior of XIV to anterior of XV. Gastropore conspicuous at XIII a1. Gastroporal duct simple, tubular. Male gonopore at XI b6, female gonopore at XIII a1, gonopores separated by 1/2 + 4 + 1/2 annuli. Small paired sperm duct bulbs in XV. Epididymis absent. Atrial cornua, coniform, undeveloped.

##### Materials examined.

Holotype. KUZ Z208, mature specimen of 70.9 mm length, dissected, collected from Yangmingshan National Park (alt. 779 m, 25°11'07"N, 121°31'10"E), Taipei City, Taiwan, by Win-Je Chi on March 24, 2011. Paratypes (a total of five specimens collected from Taiwan in 2005–2011): KUZ Z197, from Jinsan Township, Taipei County (alt. 739 m, 25°11'01"N, 121°30'54"E), on March 18, 2005; KUZ Z207, from the type locality (alt. 776 m, 25°09'49"N, 121°33'10"E) by Chi-Lun Lee and Win-Je Chi on July 30, 2010; KUZ Z209 (alt. 779 m, 25°11'07"N, 121°31'10"E), dissected, KUZ Z210 (alt. 600 m, 25°11'11"N, 121°31'10"E), dissected, from the type locality by Win-Je Chi on March 24, 2011; and KUZ Z211 from the type locality (alt. 737 m, 25°10'55"N, 121°30'50"E) by Win-Je Chi on April 24, 2011.

##### Etymology.

The specific name is taken from the native Taiwanese tribe Ketagalan. The type locality of this new species is in an area settled by this aboriginal tribe. The specific name is a native word, not a Latin or Latinized word.

##### Description of holotype.

**Figure 2.**
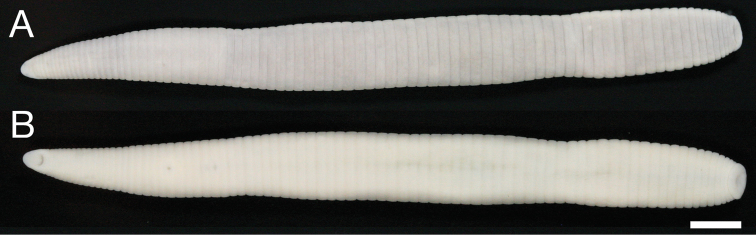
*Orobdella ketagalan* sp. n., holotype, KUZ Z208. **A** Dorsal and **B** ventral views. Scale bar, 5 mm.

Body firm, muscular, elongated, gaining regularly in width in caudal direction, dorso-ventral depressed, sides nearly parallel from mid-length to point just anterior to caudal sucker, BL 70.9 mm, BW 6.4 mm (Fig. 2). Caudal sucker ventral, oval, diameter smaller than BW (Figs 2B, 3D). Color faded in preservative (Fig. 2).

Somite I completely merged with prostomium (Fig. 3A). Somite II uniannulate, not separated from I (Fig. 3A). Somites III and IV uniannulate (Fig. 3A). Somite V biannulate, (a1 + a2) = a3, a3 forming posterior margin of oral sucker (Fig. 3A, B). Somites VI and VII triannulate, a1 = a2 = a3 (Fig. 3A, B). Somites VIII–XXV quadrannulate, a1 = a2 = b5 = b6 (Fig. 3A–E); b5 of X being first annulus on clitellum, a2 of XIII being last annulus of clitellum (Fig. 3E). Somite XXVI triannulate, a1 > a2 > a3, a3 being last complete annulus on venter (Fig. 3C, D). Somite XXVII incomplete uniannulate with slight furrow (Fig. 3C); anus behind it with no post-anal annulus (Fig. 3C).

**Figure 3. F3:** *Orobdella ketagalan* sp. n., holotype, KUZ Z208. **A** Dorsal and **B** ventral views of somites I–VIII **C** dorsal and **D** ventral views of somites XXV–XXVII and caudal sucker **E** ventral view of somites X b5–XIII **F** ventral view of gastroporal duct; and **G** ventral view of gastropore and female gonopore. Scale bars, 1 mm (**A–F**) and 0.25 mm (**G**). Abbreviations: an, anus; cl, clitellum; cp, crop; fp, female gonopore; gd, gastroporal duct; gp, gastropore; mp, male gonopore; np, nephridiopore; and ph, pharynx.

**Figure 4.**
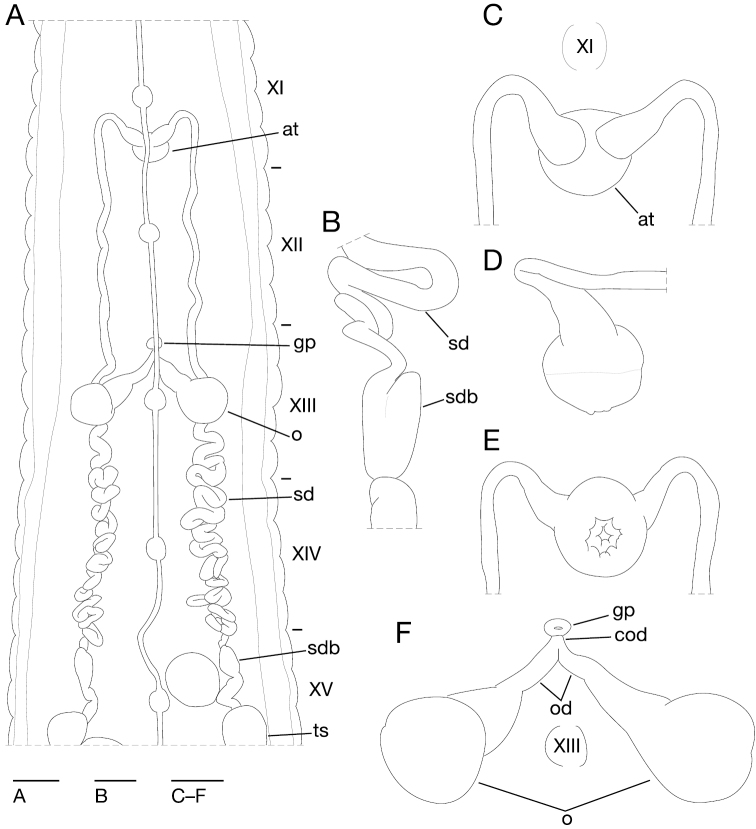
*Orobdella ketagalan* sp. n., holotype, KUZ Z208. **A** Dorsal view of reproductive system including ventral nervous system **B** lateral view of bulb of right sperm duct **C** dorsal **D** lateral, and **E** ventral views of male atrium: **C** including position of ganglion XI; and **F** dorsal view of female reproductive system including position of ganglion III. Scale bars, 1 mm (**A**), 0.5 mm (**C–F**), and 0.25 mm (**B**). Abbreviations: at, atrium; cod, common oviduct; gp, gastropore; o, ovisac; od, oviduct; sd, sperm duct; sdb, sperm duct bulb; and ts, testisacs.

Anterior ganglionic mass in VI a2 and a3. Ganglia VIII–XXI in a2 of each somite (Fig. 4A). Ganglion XIII in a2 and b5 (Fig. 4A). Ganglia XIV–XXIII in a2 of each somite (Fig. 4A). Ganglia XXIV and XXV in a1 and a2 of each somite. Ganglion XXVI in b6 of somite XXV. Posterior ganglionic mass in XXVI a1–a3.

Eyes, three pairs, first pair dorsally in furrow of II/III, second and third pairs dorsolaterally on posterior margin of V (a1 + a2) (Fig. 3A). Nephridiopores, 17 pairs, ventrally at posterior margin of a1 of each somite of VIII–XXIV (Fig. 3B, E). Papillae numerous, minute, hardly visible, one row on every annulus.

Pharynx agnathous, euthylaematous, reaching to XV a1 (Fig. 3F). Crop tubular, acecate, in XV a1 to XXI a2. Gastropore conspicuous, ventral, located middle of XIII a1 (Fig. 3E, G). Gastroporal duct narrow, simple tubular, joining with crop in XIV/XV (Fig. 3F). Intestine tubular, acecate, in XXI a2 to XXIV b5/b6. Rectum tubular, thin-walled.

Male gonopore located at middle of XI b6 (Fig. 3E). Female gonopore at middle of XIII a1, inconspicuous, located behind gastropore (Fig. 3E, G). Gonopores separated by 1/2 + 4 + 1/2 annuli (Fig. 3E). Testisacs multiple, one or two testisacs on each side in each annulus, in XV a2 to XXV b5 (Fig. 4A). Sperm ducts in XI b5 to XV a2, coiled in XIII b5 to XV a1 (Fig. 4A): small paired sperm duct bulbs in XV a1 (Fig. 4A, B). Epididymides absent. Ejaculatory bulbs absent. Paired atrial cornua in XI b5 and b6, undeveloped, coniform (Fig. 4A, C). Atrium body short, muscular, globular in XI b5 and b6 (Fig. 4A, C–E). Penis sheath and penis absent. Ovisacs, one pair, thin-walled, globular, in XIII a2 and b5 (Fig. 4A, F). Oviducts thin-walled, right oviduct crossing ventrally beneath nerve cord, both oviducts converging into common oviduct in XIII a1/a2 (Fig. 4A, F). Common oviduct thin-walled, very short, directly ascending to female gonopore (Fig. 4F).

##### Variation.

Maximum BL 111.7 mm, maximum BW 10.3 mm (KUZ Z210). In life, dorsal surface grayish, slightly darker in first third of dorsum, ventral surface whitish. Somite XXVI dorsally quadrannulate, ventrally triannulate (KUZ Z197, Z207, Z211) or quadrannulate (KUZ Z210). Somite XXVII incomplete biannulate. Pharynx reaching to XIV a1/b5–b6. Crop reaching to XXI a2/b5–XXI/XXII. Gastroporal duct joining with crop in XIV b5–XIV b5/b6. Intestine reaching to XXIV a2/b5–XXV a2. Testisacs in XV a2–XVI b6 to XXIII a1–XXV a2. Paired sperm duct bulbs in XV a1 and a2 (KUZ Z209), in XV b5 (KUZ Z210). Right or left oviducts crossing ventrally beneath nerve cord.

##### Distribution.

Known from Yangmingshan National Park and adjacent areas in northern Taipei City, Taiwan (Fig. 1).

##### Remarks.

*Orobdella ketagalan* differs from the five other quadrannulate *Orobdella* species (i.e., *Orobdella esulcata* Nakano, 2010, *Orobdella kawakatsuorum* Richardson, 1975, *Orobdella koikei*, *Orobdella tsushimensis*, and *Orobdella whitmani*) in the following combination of characteristics (Table 3): IV uniannulate, gonopores separated by 1/2 + 4 + 1/2 annuli, XXV quadrannulate, gastroporal duct simple and tubular, paired sperm duct bulbs in XV, epididymides absent, and atrial cornua undeveloped. Because *Orobdella ketagalan* possesses quadrannulate mid-body somites, this new species is easily distinguishable from the four sexannulate species (i.e., *Orobdella dolichopharynx* Nakano, 2011, *Orobdella ijimai* Oka, 1895, *Orobdella mononoke* Nakano, 2012, and *Orobdella shimadae* Nakano, 2011) and one octannulate species, *Orobdella octonaria* Oka, 1895.

**Table 3.** Comparison of morphological characters between *Orobdella ketagalan* sp. n. and five quadrannulate congeneric species.<br/>

Character	*Orobdella ketagalan* sp. n.	*Orobdella esulcata*	*Orobdella kawakatsuorum*	*Orobdella koikei*	*Orobdella tsushimensis*	*Orobdella whitmani*
Annulation of IV	uniannulate	uniannulate	biannulate	uniannulate	uniannulate	uni- or biannulate
Number of annuli between gonopores	1/2 + 4 + 1/2	2/3 + 4 + 1/3	6	1/2 + 4 + 1/2	1/2 + 5	1/2 + 4 +1/2
Annulation of XXV	quadrannulate	quadrannulate	quadrannulate	triannulate	quadrannulate	quadrannulate
Gastroporal duct	simple tubular	tubular, but bulbous at junction with gastropore	simple tubular	tubular, but bulbous at junctions with gastropore and crop	bottle-shaped	bulbiform
Paired sperm duct bulbs	in XV	absent	absent	absent	absent	absent
Epididymides	absent	XVI to XX	XVI to XVII	XVII to XIX	XVI to XIX	XVI to XVIII
Atrial cornua	undeveloped	ovate	undeveloped	ovate	coniform	ovate

## Phylogenetic analyses

**Figure 5.**
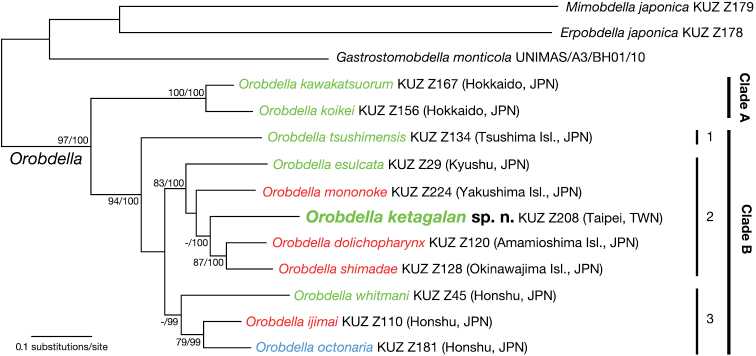
The BI tree of 3790 bp of nuclear 18S rDNA and histone H3, and mitochondrial COI, 12S rDNA, tRNA^Val^, 16S rDNA. A species name in green indicates a quadrannulate species; in red, sexannulate; and in blue, octannulate. The numbers associated with the nodes represent the bootstrap values for ML (BS) and Bayesian posterior probabilities (BPPs). BSs higher than 70 % and/or BPPs higher than 95 % are indicated. Abbreviations: JPN, Japan; and TWN, Taiwan.

The BI tree (Fig. 5) was nearly identical to the ML tree with ln *L* = -12357.61 (not shown). Monophyly of the genus *Orobdella* was well supported (BS = 97%, BPP = 100%). *Orobdella* then divided into two clades: clade A (BS = 100%, BPP = 100%) consisted of two species from Hokkaido, Japan, *Orobdella kawakatsuorum* and *Orobdella koikei*; and clade B (BS = 94%, BPP = 100%) included the other nine *Orobdella* species. Clade B was split into three subclades: subclade B1 included only *Orobdella tsushimensis* (from Tsushima Island, Japan); subclade B2 (BS = 83%, BPP = 100%) included *Orobdella esulcata* (from Kyushu, Japan), *Orobdella mononoke* (from Yakushima Island, Japan), *Orobdella dolichopharynx* (from Amamioshima Island, Japan), *Orobdella shimadae* (from Okinawajima Island, Japan), and *Orobdella ketagalan* (from Taipei, Taiwan); and subclade B3 (BS = 69%, BPP = 99%) consisted of three species (from Honshu, Japan), *Orobdella whitmani*, *Orobdella ijimai*, and *Orobdella octonaria*. Subclades B2 and B3 formed a monophyletic clade in both analyses, but with low support (BS = 67%, BPP = 89%).

In subclade B2, three species from the Ryukyu Archipelago, *Orobdella mononoke*, *Orobdella dolichopharynx*, and *Orobdella shimadae*, and the Taiwanese *Orobdella ketagalan* formed a monophyletic clade, but this clade was also not sufficiently supported (BS = 55%, BPP = 82%). Monophyly of *Orobdella ketagalan*, *Orobdella dolichopharynx*, and *Orobdella shimadae* was supported in the BI analyses (BPP = 100%), but was not recovered in the ML analyses (BS = 46%). Monophyly of *Orobdella dolichopharynx* and *Orobdella shimadae* was confirmed (BS = 87%, BPP = 100%).

## Discussion

The phylogenies obtained in this study are nearly identical to those obtained in other phylogenetic analyses of the genus *Orobdella* (Nakano 2012a, b, Nakano et al. 2012). The most ancestral clade of *Orobdella* (clade A in Fig. 5) is distributed in Hokkaido, Japan. The other species inhabit islands south of Hokkaido (clade B in Fig. 5) and are divided into three subclades (B1–3 in Fig. 5). In our analyses, however, the phylogenetic relationships of these subclades were not sufficiently resolved. Our phylogenetic trees clearly indicated that the quadrannulate mid-body somite annulation is a plesiomorphic character of *Orobdella*, and that sexannulate mid-body somites had evolved in parallel. This result was also mentioned in previous studies (Nakano 2012a, b, Nakano et al. 2012). Even in subclade B2, the sexannulate character was considered to have evolved in parallel. In this subclade, three sexannulate species from the Ryukyu Archipelago were included: *Orobdella mononoke* is from Yakushima Island, which is located in the northern part of the Ryukyu Archipelago; *Orobdella dolichopharynx* is from Amamioshima Island, which is located in the middle region of the Archipelago; and *Orobdella shimadae* is from Okinawajima Island, which is also located in the middle region of the Archipelago, but south of Amamioshima Island. Our analyses showed that these three sexannulate species did not form a monophyletic clade. In contrast, two sexannulate species, *Orobdella dolichopharynx* and *Orobdella shimadae*, and the Taiwanese quadrannulate *Orobdella ketagalan* formed a monophyletic clade. The other sexannulate species, *Orobdella mononoke*, was not closely related to *Orobdella dolichopharynx* and *Orobdella shimadae*. This is in agreement with findings by (Nakano 2012b), who mentioned that *Orobdella mononoke* was probably not very close to those two species. Our phylogenetic analyses supported his phylogenetic conclusion. According to the topologies of the ML and BI trees, *Orobdella mononoke* is a sister taxon of a clade including *Orobdella ketagalan*, *Orobdella dolichopharynx*, and *Orobdella shimadae*, but this phylogenetic position was not well resolved in either tree. To better understand the biogeographical history of *Orobdella* leeches, more robust trees for this genus based on either more DNA markers or specimens should be obtained.

*Orobdella ketagalan* possesses small, paired sperm duct bulbs in XV (Fig. 4A, B). Such small bulbs have never before been reported in *Orobdella*. Hence, small sperm duct bulbs could be considered an apomorphy of the Taiwanese *Orobdella ketagalan*. *Orobdella* species generally possess eipididymides in their male reproductive systems (Nakano 2010, 2011a, b, 2012a, b, in press). However, only *Orobdella ketagalan*, *Orobdella dolichopharynx*, and *Orobdella shimadae* do not bear epididymides (Nakano 2011b). These three species formed a monophyletic clade in our phylogenetic analyses (Fig. 5). Therefore, lacking epididymides could be considered a synapomorphy within *Orobdella ketagalan*, *Orobdella dolichopharynx*, and *Orobdella shimadae*. *Orobdella ketagalan* also possesses a simple, tubular gastroporal duct, which is similar to that of *Orobdella kawakatsuorum* (Nakano 2012a, Richardson 1975). This morphological similarity is clearly due to convergence, according to our phylogenetic analyses.

This is the first record of the genus *Orobdella* from Taiwan. Moreover, we collected several other specimens that appear to be undescribed species of *Orobdella* (Nakano and Lai, unpublished observation). Further faunal and systematic studies will reveal the species diversity of Taiwanese *Orobdella* and further elucidate the biogeographical and evolutionary history of these macrophagous leeches.

### Key to the known species of the genus *Orobdella*

**Table:** 

1	Mid-body somites more than quadrannulate	2
–	Mid-body somites quadrannulate	6
2	Mid-body somites sexannulate	3
–	Mid-body somites octannulate	*Orobdella octonaria* Oka, 1895
3	Pharynx reaching to XIV	4
–	Pharynx reaching to XVI	5
4	Gonopores separated by 1/2 + 7 + 1/2 annuli	*Orobdella ijimai* Oka, 1895
–	Gonopores separated by 8 + 1/2 annuli	*Orobdella mononoke* Nakano, 2012
5	Gonopores separated by 8 annuli	*Orobdella dolichopharynx* Nakano, 2011
–	Gonopores separated by 9 annuli	*Orobdella shimadae* Nakano, 2011
6	Color yellowish	7
–	Color grayish blue or brown	9
7	Gonopores separated by 1/2 + 4 + 1/2 annuli	8
–	Gonopores separated by 1/2 + 5 annuli, gastroporal duct bottle-shaped	*Orobdella tsushimensis* Nakano, 2011
8	Gastroporal duct bulbiform, epididymides in XVI to XVIII	*Orobdella whitmani* Oka, 1895
–	Gastroporal duct simple tubular, epididymides absent, small paired sperm duct bulbs in XV	*Orobdella ketagalan* sp. n.
9	Color grayish blue	10
–	Color brown, gonopores separated by 1/2 + 4 + 1/2 annuli	*Orobdella koikei* Nakano, 2012
10	Gonopores separated by 2/3 + 4 + 1/3, gastroporal duct simple tubular but bulbous at junction with gastropore	*Orobdella esulcata* Nakano, 2010
–	Gonopores separated by 6 annuli, gastroporal duct simple tubular	*Orobdella kawakatsuorum* Richardson, 1975

## Supplementary Material

XML Treatment for Orobdella ketagalan
